# Activation of Wnt/β-Catenin Signalling Affects Differentiation of Cells Arising from the Cerebellar Ventricular Zone

**DOI:** 10.1371/journal.pone.0042572

**Published:** 2012-08-03

**Authors:** Hayden J. Selvadurai, John O. Mason

**Affiliations:** Centre for Integrative Physiology, School of Biomedical Sciences, University of Edinburgh, Edinburgh, United Kingdom; Seattle Children's Research Institute, United States of America

## Abstract

Development of the cerebellum proceeds under the precise spatio-temporal control of several key developmental signalling pathways, including the Wnt/β-catenin pathway. We recently reported the activity of Wnt/β-catenin signalling in the perinatal cerebellar ventricular zone (VZ), a germinal centre in the developing cerebellum that gives rise to GABAergic and glial cells. In order to investigate the normal function of Wnt/β-catenin signalling in the VZ and the cell lineages it gives rise to, we used a combination of *ex vivo* cerebellar slice culture and *in vivo* genetic manipulation to dysregulate its activity during late embryonic development. Activation of the pathway at the cerebellar ventricular zone led to a reduction in the number of cells expressing the glial lineage markers Sox9 and GFAP and the interneuron marker Pax2, but had no consistent effect on either proliferation or apoptosis. Our findings suggest that activation of the Wnt/β-catenin pathway in the cerebellar ventricular zone causes a shift in the cell types produced, most likely due to disruption of normal differentiation. Thus, we propose that regulation of Wnt/β-catenin signalling levels are required for normal development of cells arising from the cerebellar ventricular zone during late embryogenesis.

## Introduction

The adult cerebellum contains a variety of types of neurons and glia, arranged in a highly characteristic laminar structure. At the centre of the cerebellum sits the white matter (WM), consisting of axonal tracts surrounding three clusters of deep cerebellar nuclei (DCN). Outside the WM sits the internal granule layer (IGL), densely populated by glutamatergic granule cells (GCs), γ-aminobutyric acid (GABA)ergic interneurons and protoplasmic astrocytes. Above the IGL sits the Purkinje cell (PC) layer (PCL), which contains the cell bodies of PCs arranged in a characteristic monolayer and interspersed with Bergmann glia (BG). PCs, GCs and Bergmann glia all extend processes into the cell-sparse molecular layer (ML), which contains a further population of interneurons (reviewed in [1], [2]).

The principal cell lineages of the cerebellum arise in a well-defined temporal manner, beginning at around embryonic day (E)10.5 in the mouse. Cells arise from two distinct germinal centres - the ventricular zone (VZ) - which lines the dorsal aspect of the fourth ventricle, and the upper rhombic lip (URL) – a transient structure at the caudal limit of the cerebellum [3]. The VZ gives rise to all cerebellar GABAergic neurons and glia, beginning with the birth of GABAergic DCN neurons at E10.5 [4], [5] and followed by the PCs, which are born in waves until E13.5 [5], [6], [7]. The VZ also generates Bergmann glia which follow the radial migration of PCs towards the pial surface [8], [9]. Interneurons and the remaining glia are then generated sequentially from the VZ and subsequently from progenitors that delaminate and continue to divide in the presumptive WM before migrating to their final positions – a process that continues into early adulthood [5], [10], [11], [12], [13]. In contrast, the upper rhombic lip gives rise to all of the glutamatergic neuron types in the cerebellum. Glutamatergic DCN neurons are specified first, from E10.5. These migrate rostrally from the URL, across the dorsal surface of the cerebellum. From E12.5, the URL gives rise to granule progenitor cells (GPCs) which stream across the pial surface of the cerebellum to form the external granule layer (EGL). The GPCs in the EGL proliferate extensively from around E18.5, continuing into the first two postnatal weeks. Following their terminal mitosis, GCs migrate inwards through the PCL to reside in the IGL – a process that is largely complete by P21 [2].

It is likely that many aspects of this developmental programme are under the control of intercellular signalling pathways. Indeed, both sonic hedgehog (Shh) and fibroblast growth factor (FGF) signalling are known to play important roles in cerebellum development [14], [15], [16], [17]. Wnt/β-catenin signalling plays a wide variety of roles at multiple stages of neural development (reviewed in [18], [19]) and is known to be required for the earliest stages of cerebellum development [20], [21]. Only recently has a developmental role for the pathway beyond this point been revealed. Pei et al. [22] identified differential effects on self-renewal, differentiation and proliferation of VZ and RL progenitors within the developing cerebellum after constitutive activation of the pathway.

Further support for a developmental function of the Wnt/β-catenin signalling pathway has been revealed from investigations into the cerebellar malignancy medulloblastoma. Activating mutations in multiple components of the pathway have been identified in tumour samples [23], [24], [25], [26], [27] and one well-defined subtype of medulloblastoma displays consistent hallmarks of Wnt/β-catenin pathway activation [28], [29], [30], [31]. While a recent study has suggested that activating mutations in the Wnt/β-catenin pathway can generate medulloblastoma from progenitors within the dorsal hindbrain [32], this does not rule out the possibility that the Wnt/β-catenin pathway could contribute to tumourigenesis from an intra-cerebellar lineage. Combined with our emerging understanding of its function during development, these observations argue that Wnt/β-catenin signalling is required for cerebellum development and that its dysregulation may contribute to the aetiology of medulloblastoma.

We recently reported a highly specific spatio-temporal pattern of Wnt/β-catenin signalling pathway activity in the developing cerebellum [33]. We showed that Wnt/β-catenin signalling is active in the ventricular zone from E18.5 and in the cell lineages radiating from this area throughout postnatal development. To investigate the potential function of Wnt/β-catenin signalling in this late embryonic/early postnatal cell population, we activated the pathway both pharmacologically in cultured cerebellum slices and by genetic activation *in vivo*. Activation of Wnt/β-catenin signalling led to a marked decrease in the production of both Sox9+ and Pax2+ cell lineages – key markers of glia and interneurons respectively. This decrease did not correlate with consistent changes in proliferation or cell death and we propose a potential role for Wnt/β-catenin signalling in regulating differentiation at the cerebellar ventricular zone.

## Results

### Wnt/β-catenin signalling is active in a subset of Sox9 expressing cells

**Figure 1:**
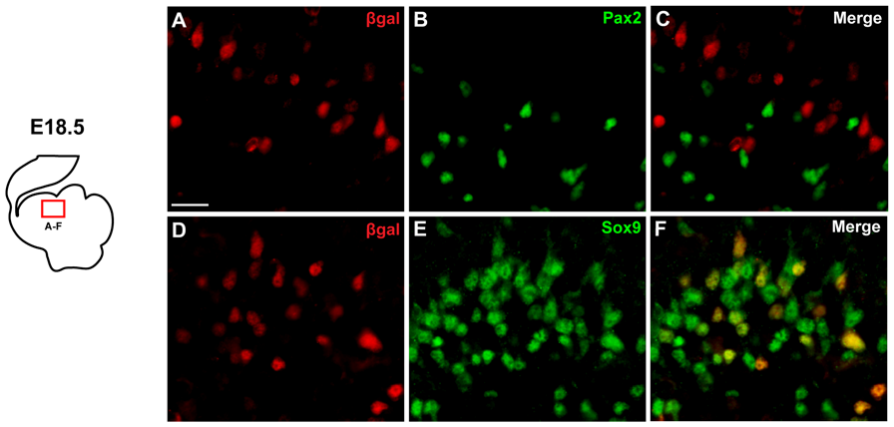
Wnt/β-catenin signalling is active in a subset of Sox9 expressing cells. Fluorescent immunohistochemistry was carried out on E18.5 sagittal BAT-gal+ cerebellum sections for β-gal (A) and Pax2 (B), but revealed no colocalisation between the two markers (C). Analysis of β-gal (D) and Sox9 (E) revealed that the clear majority of β-gal+ cells also express Sox9 (F). The boxed region in the schematic indicates the approximate position of the areas shown in all panels. Scale bar = 20 µm.

At perinatal stages, Wnt/β-catenin signalling is active in the cerebellar ventricular zone and in cells migrating radially from it [33]. At this stage the ventricular zone gives rise to two key cell types, glia and interneurons [5], and we first sought to determine in which of these lineage(s) Wnt/β-catenin signalling was active. We used mice hemizygous for the BAT-gal reporter transgene which contains a *lacZ* reporter under the control of a β-catenin/T cell factor (TCF) responsive element and is widely used as an *in vivo* reporter of Wnt/-catenin activity [34]. Using double immunohistochemistry on sections of E18.5 cerebellum from BAT-gal+ embryos, we examined the expression of the *LacZ* protein product β-galactosidase (β-gal) alongside expression of Pax2, which marks VZ-derived interneurons and their precursors [35], [36], and Sox9, which marks both the multipotent VZ and WM progenitors in addition to cells restricted to the glial lineage [37], [38], [39]. We did not find any BAT-gal+ cells that expressed Pax2 (Fig. 1A–C), indicating that Wnt/β-catenin signalling is not active in the developing interneuron lineage at this point. In contrast, the clear majority of BAT-gal+ cells expressed Sox9 (Fig. 1D–F), indicating that Wnt/β-catenin signalling at E18.5 is restricted to multipotent progenitors and/or developing glia.

### Inhibition of GSK3β activates Wnt/β-catenin signalling in cultured cerebellar slices

**Figure 2:**
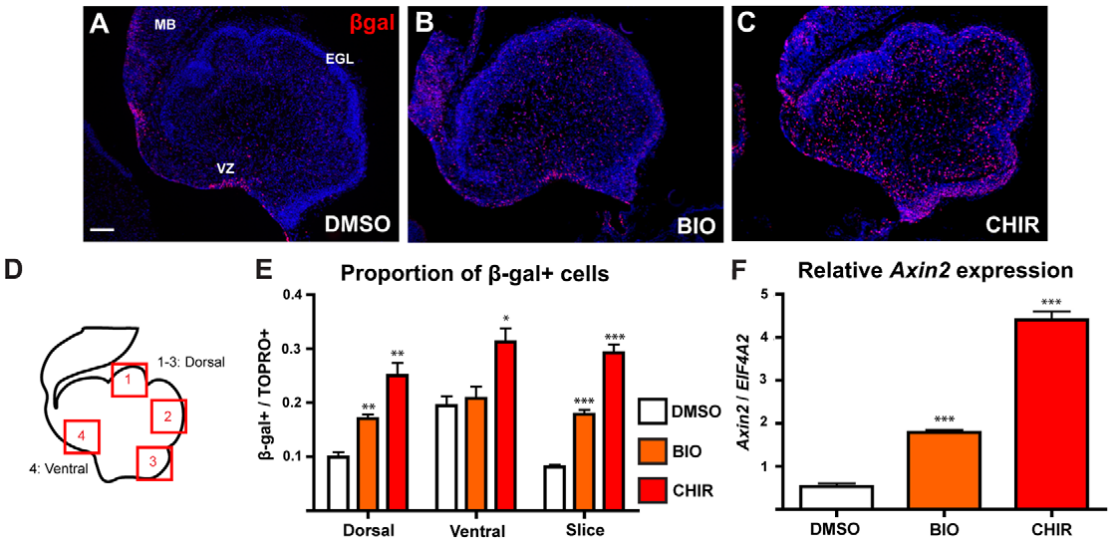
Activation of Wnt/β-catenin signalling in cultured cerebellar slices. (A–C) Immunohistochemistry showing β-gal expression in E18.5 BAT-gal+ cerebellum slices cultured in the presence of DMSO (A), 20 µM BIO (B) or 50 µM CHIR (C). (D) Schematic drawing of cerebellum slice indicating regions used for quantitation. (E) Graph showing proportion of β-gal expressing cells in specific regions of cultured cerebellum slices. Statistical comparisons were made between each of the treatment groups and the DMSO control group for each region by a Student's T-test. (F) qRT-PCR analysis of *Axin2* expression in cultured slices. Each treated group was compared to the DMSO control group by a Student's T-test. For each test, n = 3, error bars = SEM, * = p<0.05, ** = p<0.01, *** = p<0.001. Sections were counterstained with TOPRO3. Scale bar = 100 µm. EGL = external granule layer, IV = fourth ventricle, MB = midbrain, VZ = ventricular zone.

To evaluate a potential role for Wnt/β-catenin signalling in regulating aspects of cerebellum development, we first tested the effect of activating the pathway on development of the cerebellum in an *ex vivo* slice culture assay. E18.5 BAT-gal+ cerebellum slices were cultured in the presence of the GSK3β inhibitors BIO [40], [41] or CHIR [42], [43], for 24 hours. Both treatments activated Wnt/β-catenin signalling as evidenced by expression of the BAT-gal reporter (Fig. 2). The pattern of β-gal expression in control DMSO-treated slices (Fig. 2A) was identical to that observed *in vivo* [33], confirming that the culture protocol did not noticeably disrupt normal expression of the BAT-gal reporter. Treatment with 20 µM BIO resulted in increased β-gal expression (Fig. 2B), most obvious within the dorsal region including the EGL. Treatment with 50 µM CHIR caused an even more pronounced increase in BAT-gal reporter expression, with β-gal expressing cells clearly present throughout all cell layers of the slice (Fig. 2C).

To quantitate BAT-gal expression, we counted the proportion of β-gal+ cells in specific regions of each cerebellum slice, as shown in Fig. 2D. The areas indicated by boxes 1–3 (excluding the area occupied by the EGL) were designated as dorsal, and the ventral region in which cells were counted is shown by box 4. We found significant increases in the proportion of β-gal-expressing cells within the dorsal region of both BIO and CHIR treated slices compared to controls (Fig. 2E). However, only CHIR treatment resulted in a significant increase in β-gal expressing cells within the ventral region. As additional verification that treatment with the GSK3 inhibitors activated Wnt/β-catenin signalling, we measured expression of the endogenous Wnt/β-catenin target gene *Axin2* using qRT-PCR on RNA extracted from individual whole slices. Expression of *Axin2* (relative to *EIF4A2*) was significantly increased in BIO and CHIR treated slices compared to DMSO-treated controls (Fig. 2F). Taken together, these data clearly indicate that treatment with BIO and CHIR successfully activated Wnt/β-catenin signalling *ex vivo* in E18.5 cerebellum slices.

### Activation of Wnt/β-catenin signalling *ex vivo* leads to a decrease in cells expressing glial and interneuron markers

**Figure 3:**
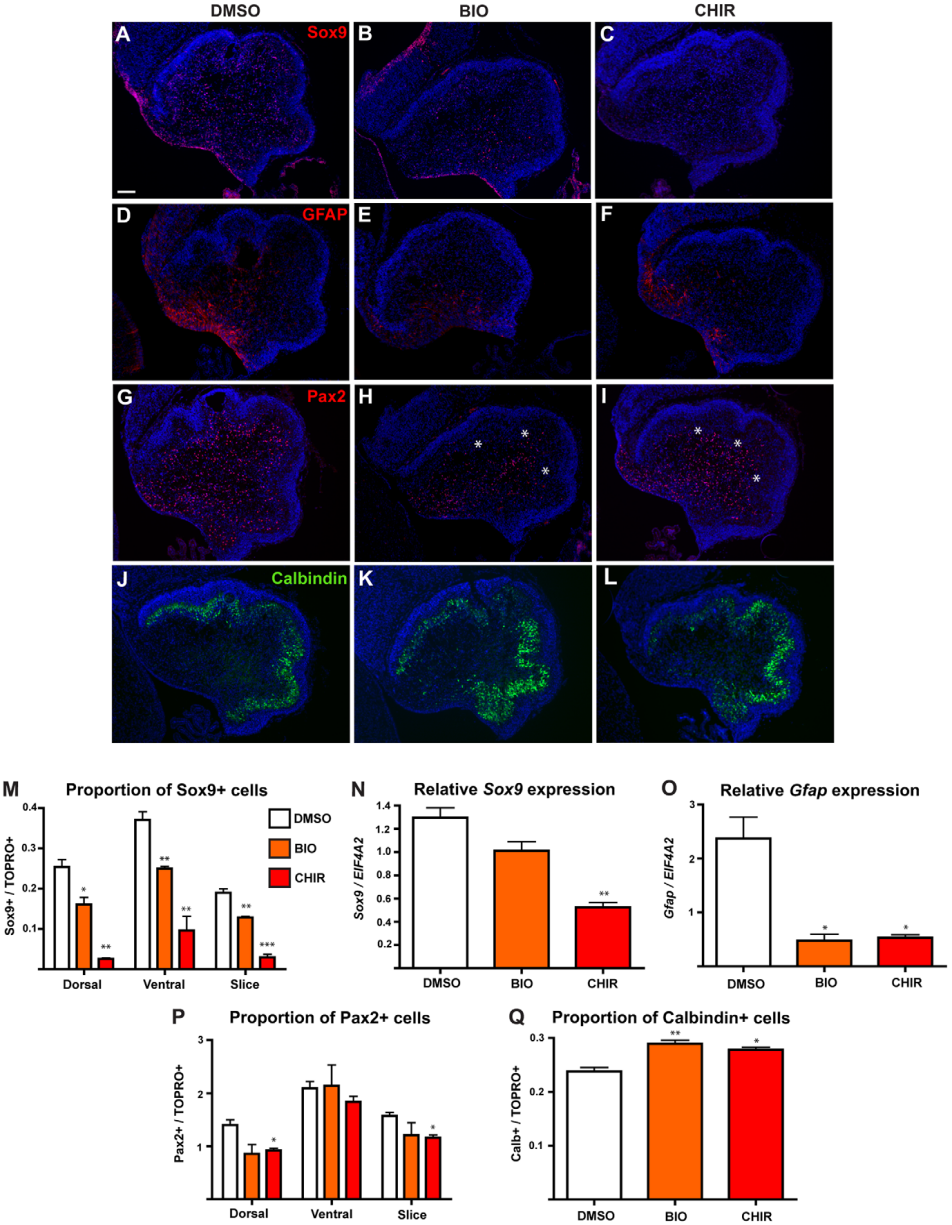
Wnt/β-catenin pathway activation *ex vivo* affects expression of VZ-derived lineage markers. (A–I) E18.5 cerebellum slices were cultured in the presence of DMSO (A,D,G), 20 µM BIO (B,E,H) OR 50 µM CHIR (C,F,I) and analysed by immunohistochemistry for the expression of Sox9 (A–C), GFAP (D–F), Pax2 (G–I) and Calbindin (J–L). (M) Graph showing the proportion of Sox9+ cells in specific regions of cultured slices. (N,O) qRT-PCR analysis of expression of *Sox9* (N) and *Gfap* (O) expression, relative to EIF4A2 control. (P) Graph showing the proportion of Pax2+ cells in specific regions of cultured slices. (Q) Graph showing the proportion of Calbindin+ cells in the dorsal region of each slice. In all cases, direct comparisons were made by comparing each treatment group with the relevant DMSO control with a Student's T-test. For each test, n = 3, error bars = SEM, * = p<0.05, ** = p<0.01, *** = p<0.001. All sections counterstained with TOPRO3. Scale bar = 100 µm.

Given that BAT-gal reporter expression in E18.5 cerebellum was seen in Sox9 expressing progenitors/glia (Fig. 1F), we first examined the effects of Wnt/β-catenin pathway activation on these cells. In DMSO treated control cultures, numerous Sox9-expressing cells were detected in both dorsal (excluding the EGL) and ventral regions (Fig. 3A), consistent with the normal pattern of Sox9 expression in VZ progenitors and their progeny [38], [39]. Treatment with BIO led to a decrease in the number of Sox9+ cells in the slice (Fig. 3B,M). An even greater decrease was seen after treatment with CHIR, with a clear loss of Sox9+ cells along the VZ (Fig. 3C,M). Quantitation confirmed a significant reduction in the proportion of Sox9+ cells within the dorsal and ventral regions of both BIO and CHIR treated slices, with CHIR producing the greatest effect (Fig. 3M). qRT-PCR analysis of *Sox9* mRNA levels in whole slices confirmed that expression was decreased in response to BIO and CHIR (Fig. 3N).

To confirm the effects of stimulating Wnt/β-catenin signalling on VZ progenitors and glia, we examined expression of an additional marker of these cell types, glial fibrillary acidic protein (GFAP) [44], [45], [46]. Immunohistochemistry on control slices revealed high GFAP expression ventrally, in particular along the VZ and in glial processes radiating from it (Fig. 3D), consistent with expression in VZ radial glia. Treatment with BIO (Fig. 3E) or CHIR (Fig. 3F) led to a considerable decrease in GFAP staining with the expression pattern becoming patchier and less intense. The extent of staining radiating from the VZ towards the dorsal region was also notably reduced. As GFAP is cytoplasmic, we were unable to quantitate cell numbers. However, the qualitative observation of GFAP immunoreactivity reduction was consistent with qRT-PCR analysis, which revealed a significant reduction in relative *Gfap* expression after treatment with both BIO and CHIR (Fig. 3O). Combined, these data indicate that activation of the Wnt/β-catenin signalling pathway at E18.5 altered the profile of progenitor/glial cells at the VZ and within the developing cerebellum.

We next investigated whether increased Wnt/β-catenin signalling affected the other cell type born in the cerebellar VZ during this developmental window, the interneuron lineage, by examining expression of the transcription factor Pax2. Pax2 is expressed in lineage restricted interneuron progenitors that arise from multipotent VZ or WM progenitors [12], [13], [36]. In control slices, Pax2+ cells were observed in a widespread pattern throughout the developing cerebellum (Fig. 3G), consistent with interneurons being generated at the VZ and radiating dorsally. This pattern of Pax2 expression was largely maintained in slices treated with BIO or CHIR, although there was an apparent restriction in the dorsal extent of Pax2 expression towards the EGL (asterisks in Fig. 3H–I). Quantitation revealed a general reduction in the number of Pax2+ cells in the dorsal region (although this difference was only significant in slices treated with CHIR), while ventrally, where most interneurons are generated at this stage, no difference was observed (Fig. 3P).

We next sought to determine the effects of constitutive Wnt/β-catenin pathway activation on the developing PC population. While these GABAergic neurons are born at the VZ and become post mitotic by E13.5, morphological maturation of the individual PCs and their development into a monolayer proceeds until the early postnatal period. Immunohistochemistry for the PC marker Calbindin [47] revealed extensive staining in the developing PCL in control slices (Fig. 3J), demonstrating that the culture system does not cause immediate disruption to the cytoarchitecture of the developing cerebellum. This pattern was largely consistent in both BIO (Fig. 3K) and CHIR (Fig. 3L) treated slices. However, quantitation of PC number revealed that in both BIO and CHIR treated slices, the proportion of Calbindin+ cells was slightly increased compared to DMSO treated slices, a finding consistent with a reduction in other cell types.

### Activation of Wnt/β-catenin signalling *ex vivo* increases proliferation but does not affect apoptosis

**Figure 4:**
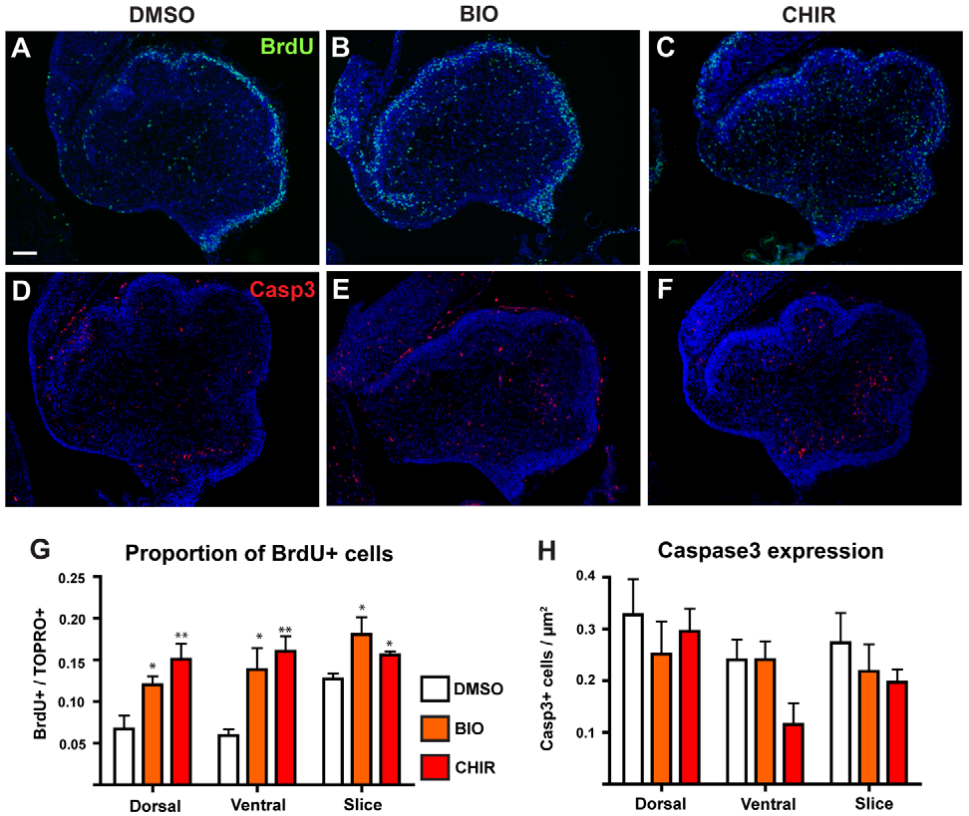
Wnt/β-catenin pathway activation *ex vivo* is mitogenic but has no affect on apoptosis. E18.5 cerebellum slices were cultured in the presence of DMSO (A,D), 20 µM BIO (B,E) or 50 µM CHIR (C,F) and analysed by immunohistochemistry for the retention of a BrdU label administered two hours prior to fixation (A–C) or apoptotic marker Caspase3 (D–F). (G) Graph showing proportion of BrdU+ cells in specific areas of cultured slices. (H) Graph showing density of Caspase3 labelled cells per unit area in specific regions of cultured slices. In all cases statistical comparisons were made by comparing each treatment group with the appropriate DMSO control using a Student's T-test. For each test, n = 3, error bars = SEM, * = p<0.05, ** = p<0.01, *** = p<0.001. All sections counterstained with TOPRO3. Scale bar = 100 µm.

One possible explanation for the decreased numbers of Sox9 and Pax2 expressing cells in cerebellum slices treated with BIO or CHIR could be reduced proliferation of progenitors. To test this possibility, we added BrdU to the cultures two hours prior to fixation and determined the proportions of BrdU labelled cells in the same defined regions of the cultured slices (Fig. 2D). With the exception of the EGL, DMSO treated control slices showed relatively few BrdU+ cells scattered throughout both the dorsal and ventral regions of the slice (Fig. 4A). Slices treated with BIO or CHIR showed an apparent increase in BrdU labelling throughout all cell layers (Fig. 4B,C). Quantitation of BrdU labelled cells supported these observations. There were significant increases in the proportion of BrdU labelled cells in the dorsal and ventral regions of both BIO and CHIR treated slices, compared to DMSO treated controls (Fig. 4G). Thus, reduced proliferation is unlikely to account for the decrease in Sox9 and Pax2 expressing cells observed in BIO and CHIR treated slices.

An alternative possible explanation for the reduction in Pax2 and Sox9 expressing cells is an increase in apoptosis. To test this possibility, we examined cultured slices for expression of the apoptotic marker Caspase3 [48]. Caspase3 labelled cells were observed throughout the control slices (Fig. 4D). Treatment with BIO (Fig. 4E) or CHIR (Fig. 4F) had no apparent effect on the number or distribution of Caspase3 expressing cells. Quantitation of Caspase3+ cells confirmed that there were no significant differences between the DMSO control and either BIO or CHIR treated slices in any of the regions analysed (Fig. 4H). Thus, the reduction in Sox9+ and Pax2+ cell numbers observed was not associated with an increase in cell death.

### *In utero* electroporation of a *cre-GFP* expression construct activates Wnt/β-catenin signalling in *Apc^lox/lox^* mice

**Figure 5:**
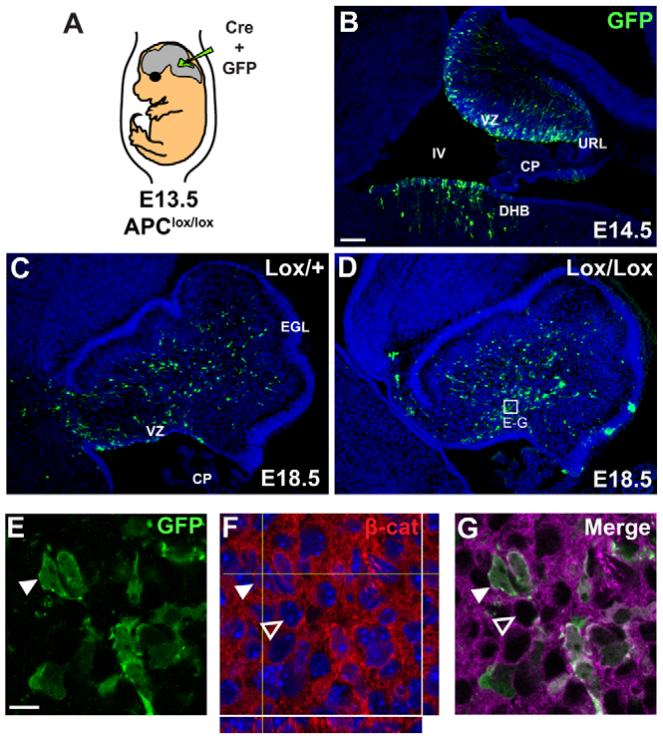
Genetic activation of Wnt/β-catenin signalling in VZ-derived cells by *in utero* electroporation. (A) Schematic illustration showing *in utero* electroporation technique. E13.5 *Apc^lox/lox^* or *Apc^lox/+^* embryos were injected *in utero* with a *Cre-GFP* plasmid delivered to the fourth ventricle and electroporated. (B) GFP expression in an electroporated embryo at E14.5, revealing extensive expression along the VZ. (C, D) By E18.5 GFP expression was widespread throughout the developing cerebellum of both *Apc^lox/+^*(C) and *Apc^lox/lox^* (D) embryos. (E–G) High magnification analysis of GFP+ cells revealed that majority of them also displayed ectopic nuclear accumulation of β-catenin (white arrowheads) compared to non-GFP cells (empty arrowheads). All sections were counterstained with TOPRO3. CP = choroid plexus, DHB = dorsal hindbrain, EGL = external granule layer, IV = fourth ventricle, URL = upper rhombic lip, VZ = ventricular zone. Scale bar = 100 µm (A–C), 10 µm (D–F).

We next sought to determine whether these effects of activating Wnt/β-catenin signalling during cerebellum development could also be observed *in vivo*. We used a strain of mice harbouring the *Apc^580s^* conditional allele of *Apc* [49] hereinafter referred to as *Apc^lox^*. In these mice, exon 14 of *Apc* is flanked by *loxP* sites and cre-mediated deletion leads to loss of functional Apc protein, thereby promoting constitutive Wnt/β-catenin activity [49]. To inactivate *Apc* specifically in the VZ, we used *in utero* electroporation to deliver a *pCAG-Cre-IRES2-EGFP* (*Cre-GFP*) plasmid [50] directly to progenitors surrounding the fourth ventricle of E13.5 *Apc^lox/lox^* embryos (Fig. 5A), the earliest stage at which VZ progenitors of interneurons and glia could be targeted reliably. Immunohistochemistry on electroporated embryos collected 24 hours later (E14.5) revealed widespread expression of GFP along the length of the VZ and in cells and processes radiating from it, indicating that cells surrounding the fourth ventricle had taken up and expressed the *Cre-GFP* plasmid (Fig. 5B). In embryos analysed at E18.5, five days post electroporation, GFP expression was much more widespread (Fig. 5C–D), indicating that electroporated cells and/or their progeny had successfully migrated through the developing tissue. No obvious differences in the distribution of GFP labelled cells were observed between *Apc^lox/+^* and *Apc^lox/lox^* embryos (Fig. 5C, D). In both cases, GFP expressing cells were evident ventrally and in a widely dispersed pattern throughout the developing cerebellum. Quantitation revealed no significant difference in the number of GFP+ cells between *Apc^lox/+^* and *Apc^lox/lox^* embryos (p = 0.309, Student's t-test, n = 4), suggesting that loss of Apc did not affect cell numbers at this stage of development.

To determine whether cre-mediated inactivation of the *Apc* ^lox^ allele led to activation of Wnt/β-catenin signalling, we examined electroporated *Apc^lox/lox^* embryos for the ectopic localisation of β-catenin to the nucleus (Fig. 5E–G). At E14.5, we found no nuclear β-catenin in any sections analysed (data not shown). However, at E18.5, a clear majority of GFP+ cells in electroporated *Apc^lox/lox^* embryos contained nuclear β-catenin (Fig. 5E–G). No nuclear β-catenin was seen in any *Apc^lox/+^* embryos (data not shown). Quantitation of randomly chosen fields in ten electroporated *Apc^lox/lox^* embryos revealed nuclear localised β-catenin in 82% (±6.2%) of GFP+ cells, indicative of active Wnt/β-catenin activity.

### Wnt/β-catenin activation *in vivo* affects the development of ventricular zone derived cell lineages

**Figure 6:**
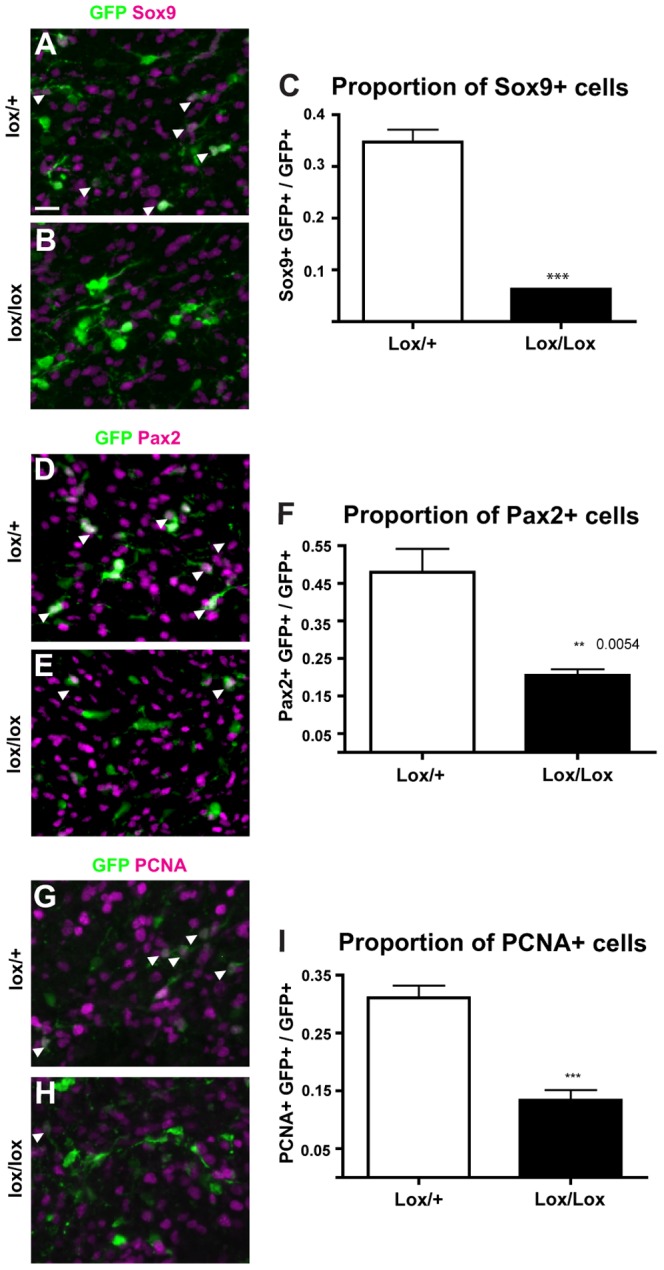
Effects of *in vivo* activation of Wnt/β-catenin signalling on development of VZ-derived cells. *Apc^lox/+^* and *Apc^lox/lox^* embryos were electroporated with a *Cre-GFP* plasmid at E13.5 and analysed at E18.5. Electroporated cells and their progeny, marked by expression of GFP, were examined for expression of Sox9 (A–C), Pax2 (D–F) and PCNA (G–I). Immunohistochemical analyses were quantitated by counting the number of GFP+ cells expressing each marker (white arrows) as a proportion of total GFP+ cell numbers per section and comparing by Student's T-test between the two genotypes for each marker. For each test, n = 4, error bars = SEM, * = p<0.05, ** = p<0.01, *** = p<0.001. Scale bar = 25 µm.

To determine the consequences of activated Wnt/β-catenin signalling at the VZ *in vivo*, we first examined expression of the VZ derived lineage markers Sox9 and Pax2 at E18.5. We used double immunofluorescence to determine the proportion of electroporated cells and their progeny (i.e. GFP+ cells) that expressed Sox9 (Fig. 6A–C). Just 6.3% (±0.2%) of GFP+ cells in *Apc^lox/lox^* embryos expressed Sox9, compared to 34.7% (±4.8%) of GFP+ cells in *Apc^lox/+^* controls (Fig. 6C). Similarly, only 20.5% (±3.4%) of GFP+ cells in *Apc^lox/lox^* embryos expressed Pax2, compared to 47.9% (±12.5%) in *Apc^lox/+^* control embryos (Fig. 6D–F). Thus, similar to our findings in cultured slices, activation of Wnt/β-catenin signalling *in vivo* led to a reduction in the proportion of Sox9 and Pax2 expressing cells arising from the VZ.

To determine whether activated Wnt/β-catenin signalling affected proliferation in this setting, we examined the expression of proliferating cell nuclear antigen (PCNA), a marker of mitotic cells (Fig. 6G–I). We found a significant reduction in the proportion of GFP+ cells that expressed PCNA in *Apc^lox/lox^* embryos (13.4%±3.5%) compared to that in *Apc^lox/+^* controls (31.1%±4.2%) (Fig. 6I). Therefore, activation of Wnt/β-catenin signalling *in vivo* during this window led to an apparent reduction in proliferation.

## Discussion

### Wnt/β-catenin signalling can be activated in the developing cerebellum *ex vivo* and *in vivo*

Following up on our previous finding that Wnt/β-catenin signalling is active at the perinatal VZ [33] we set out to investigate the normal function of this pathway in cells generated at the VZ during this developmental window. The VZ gives rise to both interneurons and glia at this time point and we first examined whether Wnt/β-catenin activity was restricted to one or other lineage. Analysis of BAT-gal Wnt/β-catenin reporter mice [34] revealed that the clear majority of cells expressing the reporter also expressed the glial/progenitor marker Sox9 [37], [38], [39] (Fig. 2F), but not the early interneuron lineage marker Pax2 [36] (Fig. 2C). This suggests that Wnt/β-catenin signalling activity is restricted to progenitor cells and/or cells entering the glial lineage, and is lost in cells that enter the interneuron lineage. Based on this observation, we hypothesised that Wnt/β-catenin signalling activity could play a role in VZ progenitor regulation or in development of the glial lineage. We therefore tested the effects of activating Wnt/β-catenin function in two experimental settings, pharmacological intervention in *ex vivo* slice culture and *in vivo* genetic manipulation via *in utero* electroporation.

We cultured slices of E18.5 BAT-gal+ cerebellum in the presence of GSK3β inhibitors BIO and CHIR. In both cases we observed a significant increase in the proportion of cells expressing the BAT-gal reporter (Fig. 2D,E,G) and the endogenous Wnt/β-catenin target *Axin2* (Fig. 2F) after 24 hours, confirming successful activation of the pathway. To complement this *in vitro* approach, and to target the VZ specifically, we also activated Wnt/β-catenin signalling at the VZ *in vivo* by electroporating a *Cre-GFP* expression construct [50] into the fourth ventricle of E13.5 *Apc^lox/lox^* embryos [49]. Analysis of GFP expression after 24 hours showed that VZ cells lining the fourth ventricle had taken up and expressed the plasmid (Fig. 2B) and by E18.5 GFP+ cells were distributed extensively through the cerebellum (Fig. 2C–D). β-catenin protein was found in the nuclei of the majority of GFP+ cells at E18.5, clearly indicating successful activation of Wnt/β-catenin signalling activity (Fig. 2E–G).

### Activation of Wnt/β-catenin signalling inhibits expression of VZ-derived lineage markers Sox9 and Pax2

Numerous lines of evidence suggest that Wnt/β-catenin signalling plays a role in regulating the proliferation, self-renewal and differentiation of radial glia within the developing cortex [50], [51], [52], [53], [54], [55], [56]. We therefore asked whether it may have a similar function in regulating development at the cerebellar VZ. The most striking effect of activating Wnt/β-catenin signalling *ex vivo* and *in vivo* was the consistent reduction in Sox9 expression in cells at, and arising from, the VZ (Fig. 3A–C, M–N and Fig. 6A–C). Analysis of a further glial/progenitor marker GFAP in the cerebellum slices treated *ex vivo* revealed a similar effect (Fig. 3D–F, O), consistent with the suggestion that reduced Sox9 expression indicates a decrease in the number of these cells, rather than a specific effect on the expression of one protein.

We also found a differential effect on generation of Pax2+ interneurons in both experimental systems. While the slices treated *ex vivo* revealed a modest reduction in Pax2 expression in cells that had migrated away from the VZ (Fig. 3G–I, P), activating Wnt/β-catenin signalling in the VZ *in vivo* caused more widespread reduction (Fig. 6D–F). This most likely reflects the different methods of pathway activation employed and the fact that in the latter, analysis was carried out a number of days after activation of the pathway while in the *ex vivo* experiment analysis was carried out after only 24 hours. However, if activation of the pathway does indeed have an inhibitory effect on normal development from the Sox9+ radial glia within the VZ, then it is not unreasonable to conclude that activating the pathway at the earlier stage in the *in vivo* experiments would cause a more profound effect on the development of the Pax2+ interneuron lineage from these progenitors. Furthermore, the reduction in Pax2+ cell number observed *ex vivo* at the periphery of each slice argues for an additional disruption during the later stages of interneuron development. While both these points now warrant further investigation, our preliminary data would suggest that Wnt/β-catenin signalling could be required at multiple stages in the development of glial and interneuron lineages from the VZ.

### Activation of Wnt/β-catenin signalling does not consistently alter proliferation and apoptosis

As Wnt/β-catenin signalling is known to be mitogenic and can promote both cell survival [57], [58], [59] and apoptosis [60], [61] depending on context [62], we investigated whether effects on these processes could underlie the observed reduction in Sox9 and Pax2 expressing cells. In the *ex vivo* cultured cerebellum slices, we observed an increase in BrdU label retention in all cell regions except the EGL after treatment with both BIO and CHIR (Fig. 4A–C, G), suggesting that activating Wnt/β-catenin activity has a mitogenic effect in this context. In contrast, the embryos analysed after targeting Apc function *in vivo* revealed a reduction in expression of PCNA in the electroporated cell population (Fig. 6G–I), suggesting a reduction in proliferation of these cells. However, the observation that the total number of electroporated (GFP+) cells did not differ between the two *Apc* genotypes argues that this reduction in proliferation within the *Apc^lox/lox^* embryos is not the result of a dramatic or sustained effect on cell cycle.

While at first these observations appear inconsistent and the different model systems employed make comparison difficult, our data suggest that activation of the Wnt/β-catenin pathway elicits a differential response depending on the developmental window during which activation takes place and the length of time allowed for an effect to become manifest. This conclusion is supported by recent findings from Pei et al. [22], who demonstrated using both *in vitro* and *in vivo* models that constitutive activation of Wnt/β-catenin signalling in VZ progenitors during embryogenesis is mitogenic initially, but this effect is not sustained and by P0 a severe effect on self-renewal and differentiation of these progenitors is observed. The conclusion that Wnt/β-catenin signalling could function differently at discrete stages in development is further supported by investigation during cortical development, where activation of the pathway in neural stem cells (NSCs) causes increased self-renewal and an expansion of the NSC population [51], while activation of the pathway in the more restricted intermediate progenitor cell population promotes differentiation and causes a depletion of the progenitor pool [52], [54].

While a precise developmental function for the Wnt/β-catenin pathway throughout the key stages of cerebellum development awaits further clarification, taken together our findings support the conclusion that activation of the Wnt/β-catenin pathway at the VZ during late embryonic development leads to a decrease in the output of Sox9, GFAP and Pax2 expressing cells from the VZ and early WM, most likely as a result of altered differentiation.

## Methods

### Ethics statement

The licence authorising this work was approved by the University of Edinburgh's Ethical Review Committee on 22nd September 2008 (application number PL35-08) and by the Home Office on 6th November 2008. Animal husbandry was in accordance with the UK Animals (Scientific Procedures) Act 1986 regulations. To minimise animal suffering, pregnant dams were culled by cervical dislocation under terminal anaesthesia according to the Code of Practice for Humane Killing of Animals under Schedule 1 to the Animals (Scientific Procedures) Act 1986 issued by the Home Office.

### Mice

The day on which the vaginal plug was detected was designated E0.5 and the day of birth as P0. BAT-gal mice were maintained as hemizygotes on a C57BL/6J background and were genotyped as described previously [34]. Mice carrying the *Apc^lox^* allele were maintained on a CBA/C57Bl/6J genetic background. Genotyping was carried out as described previously [49].

### Organotypic culture methods

*Ex vivo* organotypic slice culture procedures generally followed those of Anderson et al. [63]. E18.5 embryos were dissected in 1× Kreb's buffer on ice. Brains were removed and placed into 1× Kreb's buffer with 10 mM HEPES buffer (Invitrogen), Gentamicin (Sigma) and Penicillin-Streptomycin (Invitrogen). The cerebellum and surrounding tissue was microdissected and embedded in molten 4% LMP agarose (Seakem)/PBS at 43°C with stirring and solidified on ice. Tissue was sectioned sagitally on a vibratome at a thickness of 300 µm. Slices were collected into 1× Kreb's buffer with HEPES and antibiotics on ice. Collected Vermis slices were transferred onto polycarbonate membranes (10 mm, 8 µm pore, Whatman) floating on 2 ml of 10% FCS (Gibco) and 0.5% glucose (Sigma) supplemented MEM (Gibco) with Penicillin-Streptomycin in a 7 cm Petri dish. Slices were allowed to recover at 37°C with 5% CO_2_ for one hour before media was replaced with serum-free neurobasal medium (Gibco) supplemented with 2% B-27 (Sigma), glucose, L-glutamine and Penicillin-Streptomycin. Small molecule GSK3β inhibitors were dissolved in DMSO and added to pre-warmed Neurobasal medium+supplements at a final concentration of 20 µM for BIO (Sigma) and 50 µM for CHIR (Cambridge Biosciences). This was added to the slice cultures after they were allowed to equilibrate to the Neurobasal medium+supplements and then cultured for 24 hours. 2 hours prior to the end point BrdU was added to the medium at a final concentration of 10 mg/ml.

### In utero electroporation

E13.5 timed pregnant females were anaesthetised by inhalation with isoflurane and maintained in anaesthesia with O_2_ delivered through a facemask. Uterine horns were surgically exposed and approximately 3 µl of purified *pCAG-Cre-IRES2-EGFP* plasmid DNA [50] (>1.5 µg/µl) in H_2_O with 2 mg/ml Fast Green (Sigma) was injected into the fourth ventricle to each embryo using pulled glass capillaries inserted through the roof plate into the ventricle. A PV820 Picrospritzer (WPI) was used to deliver short bursts of plasmid solution until the Fast Green dye was visible filling the ventricle. Forceps-type electrodes (7 mm, Harvard Apparatus) were positioned around the midbrain-hindbrain boundary of embryos outside the uterine wall (anode oriented to the right) and a CUY21 electroporator (Nepa-gene) was used to deliver five pulses with the following parameters: intensity = 45 V, pulse length = 50 ms, pulse interval = 950 ms. At the end of the procedure uterine horns were repositioned in the abdomen and the smooth muscle lining was closed with grade 5 sutures and the skin was closed with 5 mm wound staples. Brains were dissected at defined stages during embryonic development and those with successful electroporations were identified by epifluorescence microscopy for GFP expression within the cerebellum.

### Histology

For *in vivo* analysis, heads were collected from E14.5 embryos and brains were dissected from E18.5 embryos. For all histological analysis, including cultured cerebellum slices, tissue was immersion fixed in 4% paraformaldehyde (PFA) in PBS overnight at 4°C. Tissue was then cryoprotected in 30% sucrose/PBS overnight before embedding in 30% sucrose/OCT (1∶1) and snap freezing. Sections were cut on a cryostat at a thickness of 12 µm for *ex vivo* slices and 14 µm for *in vivo* tissue.

### Fluorescent immunohistochemistry

Immunohistochemistry and immunofluorescence were performed according to standard protocols. Antigen retrieval was achieved by microwaving sections on medium power in 10 mM citrate buffer (pH6.0) for five minutes followed by 15 minutes at low power. Primary antibodies were rabbit anti-β-galactosidase (Molecular Probes, 1∶500), mouse anti-β-galactosidase (DSHB 1∶100), mouse anti-PCNA (Abcam, 1∶500), mouse anti-Pax2 (Covance, 1∶200), goat anti-GFP (Abcam, 1∶400), rabbit anti-GFP (Abcam, 1∶1000), rabbit anti-Sox9 (Millipore, 1∶1,000), mouse anti-β-catenin (BD Biosciences, 1∶500), mouse anti-BrdU (BD Biosciences, 1∶50), mouse anti-Caspase3 (NEB, 1∶50) and mouse anti-Calbindin (Swant, 1∶1000). All primary antibodies were detected using goat anti-rabbit IgG, goat anti-mouse IgG or donkey anti-goat IgG secondary antibodies conjugated to Alexa fluor 568 or 488 dyes (Invitrogen, 1∶200) and sections were blocked using serum from the secondary host species. Appropriate controls were used in all cases by incubating sections with all but the primary antibodies. No staining was observed under these conditions. Nuclear counterstaining was performed with TOPRO-3 (Invitrogen, 1∶1000).

### Microscopy

A Leica brightfield microscope connected to a Leica DFC360Fx camera was used to capture images of fluorescently labelled sections. Confocal microscopy for high resolution image analysis was carried out in the IMPACT Imaging Facility at the University of Edinburgh using an inverted Zeiss LSM510 confocal microscope.

### Quantitation of BAT-gal+ cells

Counting frames were placed in four defined regions of each section analysed (Fig. 2D): three along the anterior-posterior extent of the EGL, and one ventrally covering the VZ and the area immediately dorsal to it. The dorsal frames were further partitioned to exclude the EGL from the region inferior to it as the EGL contains a distinct cell population not of VZ origin.

### Quantitative gene expression analysis

RNA extraction for reverse transcriptase PCR (RT-PCR) was performed on individual snap frozen cerebellum slices using an RNeasy Microkit (Qiagen) following the manufacturer's instructions. On-column DNA digestion was performed in all cases during extraction to eliminate genomic DNA contamination. cDNA was synthesised from 50 ng RNA (measured by Nanodrop, Thermo Scientific) using a Sensiscript reverse transcription kit (Qiagen). The reactions were prepared with 50 ng sample RNA added to 1× RT-Buffer, 10 µM random hexamer primers (Promega), 0.5 mM dNTPs (Invitrogen), 10 u RNAse inhibitor (Promega), 1 µl Sensiscript reverse transcriptase and made up to 20 µl with nuclease free water. Identical reactions were set up in tandem excluding reverse transcriptase (−RT) to serve as negative controls for possible genomic DNA carryover. Reactions were carried out at 37°C for 60 minutes and stored at −20°C. Quantitative RT-PCR (qRT-PCR) was performed using a Precision qPCR SYBRgreen detection kit (PrimerDesign) and commercially synthesised primer pairs (PrimerDesign) with an Opticon DNA Engine (MJ Research) following the manufacturer's instructions. All primers used had annealing temperatures of ∼58°C and an amplicon size of 90–120 bp. Reactions were prepared in 20 µl with Precision qPCR mastermix (PrimerDesign), 300 nM custom primer pairs, 0.5 µl cDNA and made up to 20 µl with nuclease free H_2_O. Reactions were set up in a 96 well white PCR plate (Bio-Rad) using a QIAgility automated pipetter (Qiagen). H_2_O and –RT negative controls were included for each gene tested. Each reaction was begun with a 10 minute 95°C denaturation step followed by 40 cycles of: 15 seconds at 95°C, 60 seconds at 60°C. Fluorescence was monitored at each cycle and then a melt curve analysis was performed.

For each gene analysed a set of six two-fold serial dilutions of control cDNA (a pooled collection of all sample cDNAs analysed) was tested in order to generate a standard curve of arbitrarily assigned transcript units. Quantitation was carried out by comparing the relative amounts of gene-specific template cDNA to a housekeeping reference gene. The appropriate reference gene for analysis was identified using a GeNorm reference gene selection kit (PrimerDesign). This involved a test of six common reference genes by qPCR with a sample of the test cDNA followed by statistical analysis using qBase^PLUS^ (BioGazelle). This analysis determined *EIF4A2* as the optimal reference gene. Relative amounts of transcript for each gene analysed were normalised to *EIF4A2* in each case and expressed as a ratio. For every gene analysed, samples were run in triplicate using cDNA collected from three individual slices from each sample group.
